# Association of diagnostic delay with medical cost for patients with Crohn's disease: A Japanese claims‐based cohort study

**DOI:** 10.1002/jgh3.12534

**Published:** 2021-03-24

**Authors:** Eisuke Takeyama, Hiroo Wada, Setsuko Sato, Kiyohide Tomooka, Ai Ikeda, Takeshi Tanigawa

**Affiliations:** ^1^ Department of Public Health Juntendo University Graduate School of Medicine Tokyo Japan; ^2^ Department of Public Health Juntendo University Faculty of Medicine Tokyo Japan

**Keywords:** anti‐tumor necrosis factor alpha, Crohn's disease, diagnostic delay, medical cost

## Abstract

**Background and Aim:**

Longer diagnostic delay (DD) in Crohn's disease (CD) is associated with complications and related surgeries. However, the impact of DD on medical cost after CD diagnosis remains uncertain.

**Methods:**

This was a claims‐based cohort study. Our analysis used data from 2005 to 2018 from the Japanese Claims Database. We enrolled a total of 528 newly diagnosed CD patients (76.9% male) aged 31.5 ± 13.6 years. High medical cost was defined as the highest quartile of the average monthly medical cost. DD was defined as the interval between the first visit to a gastroenterologist and diagnosis with CD. In the multivariable logistic regression analysis, patients were stratified by the use of anti‐tumor necrosis factor alpha (anti‐TNFα) agents to exclude their influence on the observed effects. This study was approved by the ethics review board of the Juntendo University Faculty of Medicine (No. 2019178).

**Results:**

The multivariable‐adjusted odds ratios and 95% confidence intervals of high medical cost were 1.41 (0.81–2.43) and 0.91 (0.57–1.46), respectively, for a DD of >12 months and 1 to ≤12 months compared to <1 month. In patients receiving anti‐TNFα agents, the multivariable‐adjusted odds ratios for high medical cost were 2.63 (1.34–5.16) and 1.35 (0.79–2.28) for a DD of >12 months and 1 to ≤12 months, respectively, compared to <1 month. In patients without anti‐TNFα, multivariable logistic regression analyses were not presented because of a small number of patients categorized into the high medical cost group.

**Conclusion:**

A delayed diagnosis of CD may incur high medical cost in patients who develop aggressive disease that requires treatment with anti‐TNFα agents.

## Graphical abstract

This was a claim‐based cohort study that assessed the association between the diagnostic delay (DD) and medical cost in patients with Crohn's disease. As a result, the association between longer DD over 12 months and high medical cost defined as the highest quartile of average monthly medical cost was found in patients treated with anti‐tumor necrosis factor a agents.

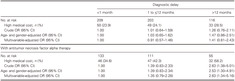

## Introduction

Inflammatory bowel disease (IBD), a chronic gastrointestinal disorder, includes primarily ulcerative colitis and Crohn's disease (CD).^1^ CD is characterized by progressive intestinal damage,^1^ which causes complications including strictures, abscesses, and fistulae, leading to intestinal surgery.^1^ The presence of perianal disease or intestinal strictures at diagnosis is a risk factor for CD with an aggressive disease course.^2^, ^3^, ^4^ To date, prevention of complications has been seen mostly as a therapeutic challenge, but it becomes clear that not only drug choice but also early timing are likely to produce better outcomes.^5^

Long diagnostic delays (DDs) among CD patients have previously been reported.^6^, ^7^, ^8^, ^9^, ^10^, ^11^, ^12^, ^13^, ^14^, ^15^, ^16^, ^17^, ^18^ In an Italian cohort study, 44% of patients with CD suffered from a DD longer than 13 months.^6^ Another cohort study conducted in Switzerland reported that 25% of patients with CD experienced DD longer than 24 months.^7^ In addition, DD is associated with CD‐related complications, including intestinal strictures and higher risk of surgery.^7^, ^8^, ^9^, ^10^, ^11^, ^12^, ^13^

Generally, CD complications cause additional treatments and contribute to a higher medical cost compared to the cost for patients without complications. To date, the impact of DD on medical cost after CD diagnosis remains uncertain. Thus, we investigated the association of longer DD with medical cost in newly‐diagnosed CD patients using data from the Japanese claims database.

Furthermore, in Japan, anti‐tumor necrosis factor alpha (anti‐TNFα) agents are authorized prescriptions for patients with steroid‐refractory and steroid‐dependent CD.^1^ In this study, we further stratified patients by their use of anti‐TNFα agents to exclude their influence on the observed effects as the efficacy of anti‐TNFα therapy was affected by disease duration,^5^, ^19^, ^20^ and use of anti‐TNFα agents led to higher health‐care costs.^21^

## Methods

### Study population

The present study was a claims‐based cohort study. We collected data from January 2005 to August 2018 from the Japanese Claims Database (JMDC Inc., Tokyo, Japan). The JMDC claims database is a large administrative claims database that includes anonymized data on inpatient, outpatient, and pharmacy health insurance claims for Japanese salaried workers and their families and is distributed by JMDC Inc., Tokyo, Japan (https://www.jmdc.co.jp/en/index). An advantage of this database is that patients can be tracked, even if the patient attends multiple medical facilities.^22^ In this database, 1967 of 4140 CD patients were newly diagnosed with CD during the study period, defined as K50 in the International Classification of Diseases, revision 10 as in a previous report.^22^ We excluded those who did not visit a gastroenterologist before CD diagnosis (*n* = 1040), were not treated by maintenance therapies for CD defined by the Japanese clinical guidelines for IBD^1^ (*n* = 283), and whose follow‐up period was shorter than 6 months (*n* = 116). A total of 528 CD patients (male: female, 406:122) were enrolled in this analysis.

### Variables

The database included age at diagnosis, gender, date of each visit to a medical institution, date of CD diagnosis and complications, type of medical institution visited (hospital or clinic), medical department, medical treatment, and medical cost. Monthly medical cost was calculated from each claim. Briefly, the average monthly medical cost was calculated from 2 months after CD diagnosis until the month of the final visit. Based on the average monthly medical cost, participants were divided into quartile groups, and the highest quartile group was defined as having high medical cost as in a previous report.^23^ The lowest quartile group was also defined as having low medical cost. The DD was defined as the interval between the first visit to a gastroenterologist and CD diagnosis and was categorized into three groups: <1 month (early diagnosis), 1 to ≤12 months, and >12 months (delayed diagnosis). The use of anti‐TNFα was defined as a prescription for anti‐TNFα (infliximab and adalimumab) after a diagnosis of CD, regardless of the treatment period and dosing amount. The follow‐up period was defined as the interval between the time of CD diagnosis and final visit and was censored when the interval between each visit was over 90 days. Complications (perianal fistulae, perianal abscesses, diarrhea, hemorrhoids, and intestinal stenosis) were also assessed based on the claims.

### Statistical analysis

To compare patient demographics according to DD category, we used an analysis of variance or Kruskal‐Wallis test for continuous variables and *χ* ^2^ tests for dichotomous variables. Age and gender, and multivariable‐adjusted odds ratios (ORs) and 95% confidence intervals (95% CIs) for high and low medical costs were determined by a logistic regression model according to DD category (<1 month, 1 to ≤12 months, and >12 months). The multivariable logistic regression model was adjusted for age at CD diagnosis, gender, follow‐up period, hospital diagnoses, hemorrhoids, perianal fistulae and abscesses, and intestinal strictures. Patients were stratified according to anti‐TNFα agent use. In additional sensitivity analyses, we defined high medical cost as the highest 10% of average monthly medical cost. High medical cost was calculated in each group for all patients and for patients treated with and without anti‐TNFα agents. All *P*‐values for statistical tests were two‐tailed, and values <0.05 were considered statistically significant. We used SAS v. 9.4 (SAS Institute, Cary, NC, USA) for all analyses.

## Results

Table 1 shows patient demographics according to the DD categories. The mean age (mean ± SD) at diagnosis was 31.5 ± 13.6 years, the mean average monthly medical cost (mean ± SD) was 163,164 ± 143,354 JPY (1,553 ± 1,365 USD), the median follow‐up period was 27 months, and the median DD was 1 month. The proportion of patients diagnosed with CD in a hospital was 86.2%. The mean age at diagnosis was higher in patients with a DD > 12 months compared with others (*P* < 0.01). The proportion of patients with perianal fistulae, perianal abscesses, and use of anti‐TNFα agents differed among DD categories (*P* < 0.05 in each case).

**Table 1:** Demographics of the patients according to diagnostic delay

	Overall	Diagnostic delay			*P*
		<1 month	1 to ≤12 months	>12 months	
Number	528	209	203	116	
Male, *n* (%)	406 (76.9)	156 (74.6)	156 (76.8)	94 (81.0)	0.42
Mean age at diagnosis, years (SD)	31.5 (13.6)	29.3 (12.4)	30.6 (14.1)	37.2 (13.1)	<0.01
Median follow‐up period, month (interquartile range)	27 (14–47)	27 (14–44)	24 (14–47)	28 (16–49)	0.59
Mean average monthly medical cost, JPY (SD)	163,164 (143,354)	167,307 (113,588)	162,941 (164,534)	156,087 (152,330)	0.80
High medical cost, *n* (%)	132 (25.0)	50 (23.9)	49 (24.1)	33 (28.5)	0.62
Low medical cost, *n* (%)	132 (25.0)	41 (19.6)	50 (24.6)	41 (35.3)	<0.01
Hospital diagnosis of Crohn's disease, *n* (%)	455 (86.2)	178 (85.2)	173 (85.2)	104 (89.7)	0.47
Complication at the diagnosis of Crohn's disease, *n* (%)					
Perianal fistula	107 (20.3)	41 (19.6)	53 (26.1)	13 (11.2)	0.01
Diarrhea	105 (19.9)	40 (19.1)	35 (17.2)	30 (25.9)	0.17
Hemorrhoid	102 (19.3)	40 (19.1)	41 (20.2)	21 (18.1)	0.90
Perianal abscess	91 (17.2)	27 (12.9)	52 (25.6)	12 (10.3)	<0.01
Intestinal stenosis	17 (3.2)	7 (3.3)	5 (2.5)	5 (4.3)	0.66
Anti‐TNFα^†^ therapy, *n* (%)	299 (56.6)	133 (63.6)	111 (54.7)	55 (47.4)	0.01

Data were tested with analysis of variance or Kruskal‐Wallis test for continuous variables and χ2 tests for dichotomous variables.

^†^ Anti‐TNFα: anti‐tumor necrosis factor alpha.

Table 2 shows the ORs and 95% CIs for high medical costs according to DD category. The ORs (95% CIs) of high medical cost for a DD of >12 months and a DD of 1 to ≤12 months were 1.41 (0.81–2.43) and 0.91 (0.57–1.46), respectively, compared to a DD of <1 month. There were 299 patients receiving anti‐TNFα, and 125 (41.8%) with high medical cost, and 229 not receiving anti‐TNFα, and 7 (3.1%) with high medical cost. After stratifying by anti‐TNFα therapy, the ORs (95% CIs) were 2.63 (1.34–5.16) and 1.35 (0.79–2.28) among patients on anti‐TNFα therapy. Multivariable logistic regression analyses were not presented for patients who did not receive anti‐TNFα therapy because there was only a small number of patients categorized into the high medical cost group. Table 3 shows the ORs and 95% CIs for low medical cost according to DD category. ORs and 95% CIs for low medical cost were 2.04 (1.18–3.52) for DD of 1 to ≤12 months and 1.39 (0.85–2.28) for DD >12 months compared to DD <1 month. In patients without anti‐TNFα agents, respective ORs (95% CIs) were 1.92 (0.91–4.04) and 1.10 (0.57–2.14). Multivariable logistic regression analyses were not presented in patients without anti‐TNFα agents because there was only one patient with low medical cost. In a sensitivity analyses, we found marginal significant association between DD >12 months and high medical cost in patients treated with anti‐TNFα agents (*P* = 0.09) (Table S1).

**Table 2:** Multivariable adjusted odds ratios (ORs) and 95% confidence intervals (CIs) for high medical costs according to diagnostic delay

	Diagnostic delay		
	<1 month	1 to ≤12 months	>12 months
No. at risk	209	203	116
High medical cost, *n* (%)	50 (23.9)	49 (24.1)	33 (28.5)
Crude OR (95% CI)	1.00	1.01 (0.64–1.59)	1.26 (0.76–2.11)
Age‐ and gender‐adjusted OR (95% CI)	1.00	1.03 (0.65–1.62)	1.47 (0.86–2.51)
Multivariable‐adjusted OR (95% CI)	1.00	0.91 (0.57–1.46)	1.41 (0.81–2.43)

With anti‐tumor necrosis factor alpha therapy			
No. at risk	133	111	55
High medical cost, *n* (%)	46 (34.6)	47 (42.3)	32 (58.2)
Crude OR (95% CI)	1.00	1.39 (0.83–2.33)	2.63 (1.38–5.01)
Age‐ and gender‐adjusted OR (95% CI)	1.00	1.39 (0.83–2.34)	2.53 (1.30–4.91)
Multivariable‐adjusted OR (95% CI)	1.00	1.35 (0.79–2.28)	2.63 (1.34–5.16)

The multivariable logistic regression model was adjusted for age at Crohn's disease diagnosis, gender, follow‐up period, hospital diagnoses, hemorrhoids, perianal fistulae and abscesses, and intestinal strictures.

For patients without anti‐tumor necrosis factor alpha therapy, multivariable logistic regression analyses were not presented because of a small number of patients categorized into the high medical cost group.

**Table 3:** Multivariable‐adjusted odds ratios (ORs) and 95% confidence intervals (CIs) for low medical cost according to diagnostic delay

	Diagnostic delay		
	<1 month	1 to ≤12 months	>12 months
No. at risk	209	203	116
Low medical cost, *n* (%)	41 (19.6)	50 (24.6)	41 (35.3)
Crude OR (95% CI)	1.00	1.34 (0.84–2.14)	2.24 (1.34–3.74)
Age‐ and gender‐adjusted OR (95% CI)	1.00	1.30 (0.81–2.09)	1.88 (1.11–3.19)
Multivariable‐adjusted OR (95% CI)	1.00	1.39 (0.85–2.28)	2.04 (1.18–3.52)

Without anti‐tumor necrosis factor alpha therapy			
No. at risk	76	92	61
Low medical cost, *n* (%)	41 (54.0)	50 (54.4)	40 (65.6)
Crude OR (95% CI)	1.00	1.02 (0.55–1.87)	1.63 (0.81–3.26)
Age‐ and gender‐adjusted OR (95% CI)	1.00	1.00 (0.54–1.87)	1.73 (0.85–3.54)
Multivariable‐adjusted OR (95% CI)	1.00	1.10 (0.57–2.14)	1.92 (0.91–4.04)

The multivariable logistic regression model was adjusted for age at Crohn's disease diagnosis, gender, follow‐up period, hospital diagnoses, hemorrhoids, perianal fistulae and abscesses, and intestinal strictures.

For patients with anti‐tumor necrosis factor alpha therapy, multivariable logistic regression analyses were not presented because of a small number of patients categorized into the low medical cost group.

## Discussion

The present study showed a significant association between longer DD and high medical cost in CD patients receiving anti‐TNFα agents. This study, to our knowledge, is the first to report that longer DD is significantly associated with high medical cost after CD diagnosis, especially among patients treated with anti‐TNFα agents. This result shows that patients who at some point require anti‐TNFα treatment for aggressive disease course are more likely to require more expensive management, including dose escalation of anti‐TNFα with longer DD. The analysis of CD patients not prescribed anti‐TNFα agents could not reveal associations between longer DD and high medical cost but was also limited by a low number of patients in the high‐cost subgroup. We found the association between DD >12 months and low medical cost. This might be because the mild patients were included in the DD >12 months group. The proportion of patients with anti‐TNFα agents in DD <1 month group were lower than in the DD >12 months group (Table 1). The mechanisms linking longer DD and high medical cost in patients receiving anti‐TNFα agents may be explained by increased health‐care utilization, that is, examinations, medications, hospitalizations, and CD‐related surgeries, due to complications caused by the DD. In several reports,^7^, ^8^, ^9^, ^10^, ^11^, ^12^ DD has been associated with further complications, and anti‐TNFα agents have been less effective for treating CD over longer disease durations compared with shorter ones,^5^, ^19^, ^20^ which may lead to additional health‐care requirements.^24^

There are several limitations in the present study. First, in previous reports, DD was defined as the interval between the onset of CD symptoms and diagnosis. In our study, we defined DD as the interval between the first visit to a gastroenterologist and diagnosis. The DDs in our study may therefore be shorter than the DDs in previous studies. A prospective study with DD defined as the interval between symptom onset and CD diagnosis is warranted. Second, misclassification is possible as we did not have the data supporting CD diagnoses, that is, endoscopy results. However, we analyzed only CD patients using maintenance therapy defined by Japanese clinical guidelines.^1^ This means the possibility of misclassification may be low. Furthermore, the diagnosis of those on anti‐TNFα agents were more likely to be assessed. Third, we did not have detailed clinical information regarding the evaluations for the severity and activity of CD, including disease location and biomarkers, because of the nature of a claims‐based study. In our study, patients were stratified by the prescription of anti‐TNFα therapy, which may indicate disease severity of CD because of the indications for anti‐TNFα agents in Japan. Fourth, in this study, the median follow‐up period of 27 months was relatively shorter than previous research. For CD patients, accumulated rates of hospitalization or surgery, which affect high medical cost, increase over time. So, the association between longer disease duration and high medical cost might be underestimated in this study because of the relatively shorter follow‐up period than in previous research.

The major strengths of our study were that confounding factors were adjusted, and we considered the influence of using anti‐TNFα agents on observed effects.

In conclusion, we showed that a delayed diagnosis of CD may incur high medical costs in patients who later require treatment with anti‐TNFα agents.

## Supporting information


**Table S1.** Multivariable adjusted odds ratios (ORs) and 95% confidence intervals (CIs) for high medical cost according to diagnostic delay.
