# Supplementary material for: Association of diagnostic delay with medical cost for patients with Crohn's disease: A Japanese claims‐based cohort study
Source: JGH Open. 2021 Mar 24;5(5):568–72. doi: 10.1002/jgh3.12534 (PMC8114978; doi:10.1002/jgh3.12534)
Supplement: Supplementary file 1 — Table S1. Multivariable adjusted odds ratios (ORs) and 95% confidence intervals (CIs) for high medical cost according to diagnostic delay. [file JGH3-5-568-s001.docx]

Table S1. Multivariable adjusted odds ratios (ORs) and 95% confidence intervals (CIs) for high medical cost according to diagnostic delay.

|  | Diagnostic delay | | |
| --- | --- | --- | --- |
|  | < 1 month | 1 to ≤ 12 months | > 12months |
| No. at risk | 209 | 203 | 116 |
| High medical cost, n, (%) | 25(12.0) | 15(7.4) | 13(11.2) |
| Crude OR (95% CI) | 1.00 | 0.59(0.30-1.15) | 0.93(0.46-1.89) |
| Age gender adjusted OR (95% CI) | 1.00 | 0.59(0.30-1.16) | 1.00(0.48-2.08) |
| Multivariable-adjusted OR (95% CI) | 1.00 | 0.57(0.29-1.13) | 0.99(0.47-2.11) |
| With anti-TNFα^†^ therapy |  |  |  |
| No. at risk | 133 | 111 | 55 |
| High medical cost, n, (%) | 11(8.3) | 9(8.1) | 10(18.2) |
| Crude OR (95% CI) | 1.00 | 0.98(0.39-2.45) | 2.47(0.98-6.20) |
| Age gender adjusted OR (95% CI) | 1.00 | 0.97(0.39-2.44) | 2.27(0.86-5.82) |
| Multivariable-adjusted OR (95% CI) | 1.00 | 0.93(0.36-2.38) | 2.38(0.88-6.48) |
| Without anti-TNFα^†^ therapy |  |  |  |
| No. at risk | 76 | 92 | 61 |
| High medical cost, n, (%) | 8(10.5) | 10(10.9) | 5(8.2) |
| Crude OR (95% CI) | 1.00 | 1.04(0.39-2.77) | 0.76(0.24-2.45) |
| Age gender adjusted OR (95% CI) | 1.00 | 0.97(0.36-2.63) | 0.65(0.20-2.13) |
| Multivariable-adjusted OR (95% CI) | 1.00 | 0.86(0.30-2.51) | 0.57(0.17-1.96) |

High medical cost was defined as the highest 10% of average monthly medical cost, and calculated in each group from all patients, and patients with and without anti-TNFα agents. The multivariable logistic regression model was adjusted for age at CD diagnosis, gender, follow-up period, hospital diagnoses, hemorrhoids, perianal fistulae and abscesses, and intestinal strictures.

OR: odds ratio, CI: confidence interval, ^†^Anti-TNFα: anti-tumor necrosis factor alpha.
